# New Insight Into the Evolutionary Arms Race Between Spider Egg Sac Pseudoparasitoids and Active Maternal Care by the Spiders

**DOI:** 10.1002/ece3.73581

**Published:** 2026-04-30

**Authors:** Agata Kostro‐Ambroziak, Urszula Suprunowicz, Alicja Piotrowska‐Niczyporuk, Magdalena Czajkowska, Elżbieta Sandurska, Dawid Szymański, Dominik Szymański

**Affiliations:** ^1^ Division of Biodiversity and Behavioural Ecology, Department of Zoology and Genetics, Faculty of Biology University of Bialystok Białystok Poland; ^2^ Doctoral School University of Bialystok Białystok Poland; ^3^ Laboratory of Plant Biochemistry, Department of Biology and Plant Ecology, Faculty of Biology University of Bialystok Białystok Poland; ^4^ Department of Zoology and Genetics, Faculty of Biology University of Bialystok Białystok Poland; ^5^ Faculty of Biological Sciences Kazimierz Wielki University in Bydgoszcz Bydgoszcz Poland; ^6^ Dawid Szymański Ślesin Poland; ^7^ Dominik M. Szymański Ślesin Poland

**Keywords:** coevolution, Darwin wasps, fatty acids (FAs), feeding of pseudoparasitoid larva, oviposition decision, reproductive effort, wolf spiders

## Abstract

Pseudoparasitoids can lead to high mortality in spider egg sacs, and in some cases, they reduce the reproductive success of a spider female to zero. On the other hand, a species that develops within the spider's egg sac uses a limited resource derived from a single egg sac for its larval development. Therefore, the most crucial behaviour that increases the fitness of free‐living pseudoparasitoid females is choosing the best host for their offspring. We analysed various points of the counter‐adaptations of the spider egg sac pseudoparasitoid and spiders exhibiting active maternal care, utilising the ichneumonid *Hidryta fusiventris* (Thomson, 1873) and the wolf spider *Pardosa lugubris* (Walckenaer, 1802). We showed that the oviposition decision of *H. fusiventris* is based on the spider's egg sac size and that the females of *P. lugubris* invest the most in the first egg sac—their relative reproductive effort is the highest at that time. We observed that there is a time shift of approximately 1 month between the first egg sac of *P. lugubris* and the presence of *H*. *fusiventris* in its egg sac, which can significantly impact *P. lugubris* fitness (tenable escape of the spider from the pseudoparasitoid). However, in the case of parasitisation, females of *P. lugubris* continue taking care of these egg sacs despite the absence of spider offspring and changes in the shape, weight and texture of the egg sacs with *H. fusiventris* pupae. This care is assumed to be, for many reasons, necessary for the proper development of this pseudoparasitoid. We observed that *H. fusiventris* larvae are able to feed on both the eggs and juveniles of spiders. They serve as long‐term dynamic food sources; however, changes in chemical composition throughout the developmental stages of *P. lugubris* may affect the development of *H. fusiventris* and its fitness‐related traits.

## Graphical abstract

We analysed various points of the counter‐adaptations of the spider egg sac pseudoparasitoid and spiders exhibiting active maternal care, utilising the ichneumonid *Hidryta fusiventris* and the wolf spider *Pardosa lugubris* . We observed that there is a time shift of approximately 1 month between the first egg sac of *P. lugubris* and the presence of *H. fusiventris* in its egg sac (tenable escape of the spider from the pseudoparasitoid). We showed that the oviposition decision of *H. fusiventris* is based on the spider's egg sac size and that *H. fusiventris* larvae are able to feed on both the eggs and juveniles of spiders. Furthermore, females of *P. lugubris* continue taking care of these egg sacs despite the absence of spider offspring and changes in the shape, weight and texture of the egg sacs with *H. fusiventris* pupae.

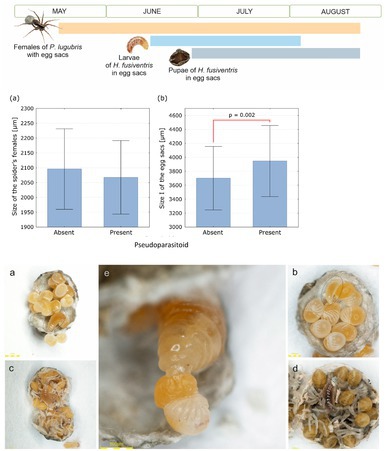

## 1 Introduction

Spider egg sacs contain valuable food resources (Anderson 1978; Laino et al. 2011, 2013; Trabalon et al. 2018) and many animal taxa specialise in feeding on spider egg masses (Durkin et al. 2021; Gudin et al. 2024; Jabłońska and Kostro‐Ambroziak 2023; Jiménez‐Conejo et al. 2024). Among them are species which develop within a single spider egg (Debnath et al. 2024) and species that develop in the spider's egg sac and feed on its content (Finch 2005; Kostro‐Ambroziak et al. 2020). Some authors both above groups treat as parasitoids of spider eggs and egg sacs, respectively (Fei et al. 2023). However, larvae that feed on multiple spider eggs do not meet the strict definition of a parasitoid (Godfray 2019) then are rather considered as egg predators (Durkin et al. 2021; Gudin et al. 2024; Quicke 2015; Souza‐Santiago et al. 2023) or egg sacs/egg masses pseudoparasitoids (Österman et al. 2026; Takasuka and Broad 2024). In our study, we concentrate on egg masses eaters and adopt the latter term (pseudoparasitoids) for them, which is used especially in relation to Ichneumonidae (Darwin wasps), where ovipositing in spider's egg sacs and larvae feeding on spider's egg masses is considered as an intermediate form to true parasitoids (koinobiont ectoparasitoids) of spiders (Matsumoto 2016).

Spiders can suffer high mortality rates from egg sac pseudoparasitoids—up to 60% in one population, and in some cases, the reproductive success of a spider female in a parasitised egg sac is reduced to zero (Finch 2005; Wawer and Kostro‐Ambroziak 2016). Thus, traits that enable avoidance of, or reduction in, egg sac parasitisation are critical for spider fitness. On the other hand, the eggs from a single egg sac are the only resource available for the pseudoparasitoid larvae to consume for their full larval development (Quicke 2015). Thus, pseudoparasitoids are also subject to strong natural selection, which in turn leads to the optimal exploitation of their hosts (Harvey 2005). Therefore, the most important behaviour that increases the fitness of free‐living pseudoparasitoid females is their oviposition decision, including the choice of a host which should be of high nutritional quality and have sufficient food resources for their offspring. All these factors exert reciprocal selective pressures on spiders and pseudoparasitoids, leading to counter‐adaptations (coevolution), resembling the concept of an arms race (Dawkins and Krebs 1979).

In general, parasitoids can exhibit remarkably high conversion rates of the energy available in their food, e.g., between 80% and 84% for the ichneumonid wasp *Diadromus pulchellus* Wesmael, 1845, which develops in the pupae of the moth *Acrolepiopsis assectella* (Zeller, 1839) (Rojas‐Rousse and Kalmes 1978). However, dietary efficiency in some spider egg sac pseudoparasitoids may be lower as it reaches only from 16% to 31% for the *Hidryta sordida* (Tschek, 1871) feeding on eggs of the wolf spider *Pardosa lugubris* (Walckenaer, 1802) (Edgar 1971). Additionally, the number of eggs in a spider egg sac differs notably inter‐ and intraspecifically (Bowden and Buddle 2012; Marshall and Gittleman 1994). Furthermore, some spiders reproduce as early in the season as possible, when adult pseudoparasitoids are absent in the habitat because some pseudoparasitoids oviposit only in fresh egg sacs and/or by spider juveniles hatch in egg sacs (Eason et al. 1967; Leborgne and Pasquet 2005; Rollard 1987). It has been recorded that pseudoparasitoid larvae do not feed on moving spiderlings (Iwata 1963). However, a few data indicate that they consume the eggs and spiderlings in the egg sac (Austad and Thornhill 1986).

The chemical composition of the successive developmental stages of the spider in the egg sac changes (Laino et al. 2013; Romero et al. 2022; Suprunowicz, Piotrowska‐Niczyporuk, and Kostro‐Ambroziak 2025; Trabalon et al. 2018). In turn, if a larva of pseudoparasitoid in spider's egg sac, feeds not only on eggs, this chemical transformation can influence its development. It is especially important in relation to essential nutrients that cannot be synthesised by parasitoids, including some ichneumonid wasps (Scheifler et al. 2024). One class of essential nutrients is found among fatty acids (FAs), which are fundamental components of lipids (Malcicka et al. 2018). The FAs serve multiple important roles, such as: membrane fluidity regulation; precursor of waxes, pheromones, eicosanoids and defence secretions; energy storage and participation in metabolic processes; development and reproduction (Kaczmarek and Boguś 2021; Stanley‐Samuelson et al. 1988). In general, when more lipid can be carried over from the host, the eater has more energetic reserves available for allocation into fitness‐related traits such as fecundity, egg composition, longevity, etc. (Scheifler et al. 2024; Visser et al. 2023). Parasitoid larvae often mimic the fatty acid composition of their host (Thompson and Barlow 1974). This means that the fatty acid profile of the parasitoid closely resembles that of the host it develops on, as parasitoids incorporate host‐derived fatty acids directly into their own fat stores rather than synthesising fatty acids *de novo*. The synthesis of fatty acids is an energy‐expensive process (Kaczmarek and Boguś 2021; Wakil 1961). It is known that some parasitoids exhibit a plasticity in fatty acid synthesis that depends on the quality of the host, while others synthesise fatty acids or lack fatty acid synthesis (Ruther et al. 2021; Visser et al. 2021).

Some pseudoparasitoids of spider egg sacs have narrow host ranges, but some are not specialised (Broad et al. 2018). Specialisation may be especially crucial for pseudoparasitoids that oviposit in egg sacs actively protected by the mother. Some spiders conceal egg sacs and stay near and guard them for several days or even until spiderlings emerge (Cuff et al. 2022; Leborgne and Pasquet 2005), while others exhibit maternal care by carrying the egg sac with their chelicerae (Austad and Thornhill 1986) or attaching it to their spinnerets (Suprunowicz, Kostro‐Ambroziak, et al. 2025). The circumvention of each distinct form of maternal defence likely requires its own specialised strategy, making it difficult for a single pseudoparasitoid to evolve multiple ones. In Lycosidae (wolf spiders), which are ground‐hunters, the female attaches the egg sac to her spinnerets with a strand and carries it with her everywhere, taking care of it (Foelix 2025). She exposes the egg sac to sunlight to optimise the temperature for egg and spiderling development, and manipulates the egg sac using the pedipalps and legs (Ruhland et al. 2016). This complex parental care is circumvented by *Gelis micrurus* (Forster, 1850), *Hidryta sordida* and *Idiolispa analis* (Gravenhorst, 1807), belonging to Ichneumonidae (Finch 2005; Schwarz and Shaw 1999). Interestingly, in some populations of wolf spider species of *Pardosa* C.L. Koch, 1833, *G. micrurus* is very common, while in others it is not found, but *H. sordida* occurs (Finch 2005).

The degree of parasitoidism of wolf spider egg sacs varies between species and populations from 0% to 51.5% (Bowden and Buddle 2012; Edgar 1971; Koltz et al. 2019). It was found that parasitoidism by *Gelis* sp. was highest in the largest species ( *P. sodalis* Holm, 1970) out of three co‐occurring species of *Pardosa*, and in the two smaller species ( *P. lapponica* (Thorell, 1872) and *P. moesta* Banks, 1892), the incidence of egg sac parasitoidism increased with spider body size (Bowden and Buddle 2012). However, the presence of *G*. *micrurus* in the *P. glacialis* (Thorell, 1872) egg sacs was not significantly related to spider female body size (Koltz et al. 2019).

Active maternal care in *Pardosa* species is considered to be triggered by mobile juveniles inside the egg sac, which can produce vibrations that stimulate the female's care (Ruhland et al. 2016, 2019). Furthermore, chemical signals are probably emitted from the egg sac inside to ensure contact between the offspring and the mother (Ruhland et al. 2017, 2019). The wolf spider female recognises its own egg using complex information such as its mass, size, shape and odour (Culley et al. 2010; Ruhland et al. 2019). Hence the question is what about an egg sac with a pseudoparasitoid? The egg sacs of *Pardosa* that contain the pupae of *H. sordida* exhibit a modified appearance (Edgar 1971); however, the presence of *G*. *micrurus* did not lead to egg sac deformation (pers. observation).

It should be emphasised that the counter‐adaptations of spiders that exhibit active maternal care and the pseudoparasitoids of spider egg sacs are poorly understood. Many issues taking into account both the oviposition decision of the pseudoparasitoid female and the feeding of the pseudoparasitoid larva in the egg sac, as well as the female spider's behaviour while taking care of the egg sac with the pseudoparasitoid in it are unknown. Thus, the aim of this study was to take a deep dive into the evolutionary arms race of spider egg sac pseudoparasitoids and the active maternal care of wolf spiders, utilising the ichneumonid wasps *Hidryta fusiventris* (Thomson, 1873) and the wolf spider *Pardosa lugubris* (Walckenaer, 1802), by testing the following hypotheses: (H: 1) pseudoparasitoids preferentially parasitise larger egg sacs; (H: 2) pseudoparasitoid larvae feed not only on spider eggs but also on the later developmental stages of spiders in the egg sac; (H: 3) spider eggs are better resources of fatty acids for pseudoparasitoid larvae than spider juveniles; (H: 4) the weight of an egg sac with pseudoparasitoid pupa does not differ significantly from the weight of an egg sac with spider offspring; (H: 5) wolf spider females do not recognise the presence of a pseudoparasitoid pupa in egg sac, and do not reject this egg sac.

## 2 Materials and Methods

### 2.1 Field Studies

Research was conducted in three locations in Poland. The first population (KAM) was found in Białystok (NE Poland), near the Campus of the University of Bialystok (53°6′24.854″ N 23°9′14.23″ E) around the artificial pond. The spiders were observed in the hollow of the land, on small stones (periodically flooded with water) and in low grass. The second population (MIC) occurred on the outskirts of Białystok city (53°5′37.914″ N 23°11′37.229″ E). It was a post‐felling area, in the close vicinity of the forest, well exposed to sunlight, dry and covered with low vegetation. The third population (KON) was located along the discharge canal of the power plant, near the village of Kolebki (52°21′27.3″ N 18°21′24.0″ E) in central Poland. Due to the temporary drying of the canal, spiders were also collected in the bed.

The study was conducted every 1–2 weeks from mid‐May to the end of August; during this time, females carrying egg sacs were present in the field. Approximately 20 females of *Pardosa* (cf. *lugubris*) with egg sacs were collected from each studied population during each visit (between 8.00 and 11.00 a.m.). Spiders were grabbed and kept separately into 60 mL containers (the survival method). The KAM populations were studied in 2018 and 2019, the MIC population in 2019, 2020 and 2023, and the KON population in 2023 and 2024. The additional material (9 parasitised egg sacs of *P. lugubris* —4 with larvae and 5 with pupae of pseudoparasitoid) was collected in south Poland in Ruda Śląska city (50°17′13.087″ N 18°52′52.872″ E) in 2023, during a study carried out by Mateusz Glenszczyk. The population (KAT) was located on the outskirts of the forested area of Dworski Park.

### 2.2 Laboratory Analysis

#### 2.2.1 Rearing and Measurements

The females with egg sacs from the KAM and MIC populations were studied in the laboratory within a period of 1–2 h after collection in the field, whereas those from the KON population were studied within 24 h due to the distance from the field to the laboratory. The egg sac was carefully detached from the female with sterile tweezers. Spider females and egg sacs were weighed using a WPA 60/C/1 (RADWAG) laboratory balance and photographed using an Olympus DSX 110 microscope. Then each egg sac was opened along the seam with sterile tweezers, and its content was analysed—the stages of development (according to Trabalon et al. (2018)) and the number of the spiders' offspring were determined. The body size of the female was defined by the maximum width of the prosoma (Bowden and Buddle 2012) and the size of the egg sac as its maximum length (size I) and width (size II). The measurements were made by using an Olympus DSX 110 microscope. Spiders were determined based on Nentwig et al. (2026), and confirmed specimens of *P. lugubris* (Walckenaer, 1802) and their egg sacs were included in the further analysis (*n* = 757).

The egg sacs with pseudoparasitoid larvae (*n* = 29) were placed into sterile Eppendorf tubes with holes in the cap. The tubes were stowed in glass containers with vermiculite pellets or coconut fibres, which were sprinkled with water every few days. Breeding was conducted at 21°C under natural light:dark (L:D) conditions. The pseudoparasitoid larvae were inspected every 1–2 days, assessing their condition, colour, size and mobility by direct observation under a microscope (larvae remained in Eppendorf tubes). From the material studied in 2019, we selected large pseudoparasitoid larvae (*n* = 5) to check their food preferences. The fresh eggs and juveniles I of other conspecific spider females were added to native egg sacs.

Some of the egg sacs with pseudoparasitoid pupae (*n* = 27) were kept for rearing in the same conditions as those with larvae. The egg sacs with pupae collected at the end of August 2019 (*n* = 15) were kept at 2°C for several weeks, according to the suggestion that the winter generation of a pseudoparasitoid should be maintained at a lower temperature to develop further (Edgar 1971). The remaining egg sacs with pseudoparasitoid (*n* = 12) were used to study the parental care behaviour of *P. lugubris* females. In both tests, spider females were kept in glass terraria with soil from the habitat where they were collected. Approximately an hour after detaching the native egg sac, the egg sac with pseudoparasitoid pupa was placed in each terrarium. In the first test, the native egg sacs (*n* = 5) were returned. Whereas in the second test, the egg sacs with eggs or juveniles were detached from *P. lugubris* females, and an egg sac with a pseudoparasitoid pupa (*n* = 7) from a conspecific female was placed in breeding containers. Acceptance or non‐acceptance of native and non‐native egg sacs and the time taken to care for the egg sac with the pseudoparasitoid pupa were observed. Every 2 days, the spiders were fed *ad libitum* with juvenile crickets ( *Acheta domesticus* Fabricius, 1775), and the terrarium substrate was sprinkled with water. The remaining breeding conditions were the same as those with pseudoparasitoid larvae and pupae.

The spider females and egg sacs that contained only spider offspring, as well as all pseudoparasitoids that emerged from the egg sacs as imago or failed in development, were put into Eppendorf tubes and left at −20°C. Adult specimens of pseudoparasitoids were determined based on morphological features (Schwarz 2005), and two of them (female and male) were also included as internal control in genetic analysis. The larvae and pupae of pseudoparasitoids were determined using molecular methods.

Our research conformed to legal requirements and guidelines established for the treatment of animals in research using invertebrate species and for their care using accepted ethical laboratory standards. The species used for the experiments are not endangered or protected in Europe.

#### 2.2.2 Molecular Analysis

Pseudoparasitoids (*n* = 58) recorded in 58 egg sacs of *P. lugubris* collected in four populations: MIC (*n* = 41), KON (*n* = 11), KAT (*n* = 5), KAM (*n* = 1) (detailed information about population—see ‘Field Studies’) were identified by molecular analysis. DNA was obtained from the hind leg in the case of imagines and from the whole individual in the case of larvae and pupae. Extraction was performed with the DNeasy Blood and Tissue Kit (Qiagen, Hilden, Germany) according to the manufacturer's instructions. Universal primers for barcoding mtDNA COI gene (Ratnasingham and Hebert 2007), LepF1 (ATTCAACCAATCATAAAGATATTGG), and LepR1 (TAAACTTCTGGATGTCCAAAAAATCA) were used for the fragment gene amplification (648 bp). PCRs were carried out in 5 μL volumes, and the reaction mixtures consisted of 2 μL of DNA, 1.7 μL of Qiagen Multiplex PCR Master Mix 1× (Qiagen, Hilden, Germany), 0.3 μL of primer mixture (0.2 μM of each primer), and 1 μL of RNase‐free water. The polymerase chain reaction cycling scheme was as follows: 15 min at 95°C followed by 40 cycles of 30 s at 94°C, 90 s at 52°C, 60 s at 72°C, and the final extension step of 30 min at 60°C. The PCR products were enzymatically purified using the EPPiC kit (A&A Biotechnology, Gdańsk, Poland) and sequenced in both directions with the BigDye Terminator v3.1 Cycle Sequencing Kit (Applied Biosystems, Foster City, CA, USA). The reaction conditions were as follows: 25 cycles with denaturation at 96°C for 20 s, annealing at 50°C for 15 s, extension at 60°C for 60 s. Sequencing reaction products were purified with the ExTerminator kit (A&A Biotechnology, Gdańsk, Poland) and separated on a 3130 Genetic Analyser (Applied Biosystems, Foster City, CA, USA). The DNA sequences were aligned in BioEdit v 7.0.4.1 (Hall 1999), revised manually for polymorphic site detection, and compared to the BOLD Identification System (https://v3.boldsystems.org). We calculated the number of haplotypes (Nh) and the number of polymorphic sites between the described haplotype using DnaSP v.5.10, and the software packages ARLEQUIN v.3.5.1.2 (Excoffier and Lischer 2010). To test the phylogenetic relationships among the *COI* mtDNA haplotypes derived in this study and sequences of the genus *Hidryta* Förster, 1869 downloaded from BOLD Systems and GenBank (https://www.ncbi.nlm.nih.gov/), we constructed a phylogenetic tree using a maximum‐likelihood (ML) algorithm in Mega v.11.0.13 (Tamura et al. 2013) with 1000 bootstrap replicates used to assess the support for tree nodes. In the phylogenetic analyses, Tamura 3‐parameter substitution model (has Invariant sites) was used as suggested by jModelTest v. 2.1.10 (Darriba et al. 2012). The tree has been rooted with mt*COI* sequences of *Trychosis* Förster, 1869 (GenBank no. KY998803) belonging to the same subtribe Agrothereutina as the genus *Hidryta*.

#### 2.2.3 Chemical Analysis of the *P. lugubris* Egg Sac Content

For the determination of FAs, proteins and monosaccharides in different developmental stages of *P. lugubris* , three types of samples were prepared: with eggs (from eight egg sacs), juveniles I (from five egg sacs), and juveniles II (from five egg sacs), respectively.

##### 2.2.3.1 Determination of Fatty Acids

All the chemicals and solvents used for GC–MS analyses were of HPLC grade from Merck (Merck KGaA, Darmstadt, Germany). The mixture of standards of fatty acids methyl esters (FAMEs) (Supelco 37 Component FAME Mix) was bought from Sigma‐Aldrich (Merck KGaA, Darmstadt, Germany). For the standards, a mixture of 100 mg of 37 FAMEs, individually varied between 2 and 6 mg, was dissolved in hexane with nonadecanoic acid (C19:0) as internal standard (IS).

The samples of proper eggs, juvenile I and juvenile II were extracted with 0.5 mL of hexane and 0.25 mL of chloroform in the presence of 0.25 mL of 1% methanol‐potassium hydroxide mixture as a catalyst in a transesterification reaction to obtain FAMEs (Van Gerpen and Knothe 2010). Thus, each material sample, three stainless steel balls, and 1 mL of organic solvents (hexane, chloroform and 1% KOH in methanol) were transferred to Eppendorf tubes for homogenisation in a ball homogeniser for 5 min at an oscillation of 50 Hz (TissueLyser LT; Qiagen GmbH, Düsseldorf, Germany). Then, the homogenised samples were kept in an ultrasonic cleaning bath (Sonorex Digital 10P, Germany) at 60°C for 90 min for simultaneous lipid extraction and transesterification reaction for fatty acid analysis. During this one‐step reaction, FAMEs were formed. After cooling, 2 mL of hexane and 1 mL of 6 M HCl were added to stop the transesterification reaction. Then the mixture was centrifuged (5 min at 4000 RPM) to separate the aqueous layer from the organic layer. The organic layer was transferred to new tubes and evaporated to dryness overnight (at 45°C). The residue was redissolved in 50 μL of hexane, and 1 μL of this solution was taken for GC–MS analysis. The FAMEs were analysed by a gas chromatograph (7890B GC System) with a mass selective detector MSD5977A (Agilent Technologies, USA). The samples (1 μL) were injected via an Agilent 7683 Injector and Sample tray Series with a split ratio of 30:1. The injector and transfer line temperatures were kept at 260°C. A Select HP‐88 capillary column (100 m × 0.25 mm, 0.20 μm, 5 in. cage) (Agilent Technologies, USA) and helium as carrier gas (1 mL/min) were used. The GC temperature program started at 140°C (with a hold time of 5 min) and was increased to 240°C at a ramp rate of 4°C/min. The electron energy was 70 eV, and the ion source temperature was set to 250°C. Quantification of data obtained from single ion monitoring (SIM) mode measurements was performed using the peak area ratios relative to that of the IS. Retention times (RT) and fragment ions, including m/z 55.1, 67.1, 79.1, 74.1, 81.1, 87.1 and 99.1 for FAMEs were recorded throughout the run. Three replicates were performed for each pooled sample.

The result obtained from each single analysis was converted into the content expressed in micrograms of fatty acid/1 g of sample [μg/g]. The mean and standard deviation (SD) were calculated considering all replicates of each sample. The relative proportion [%] of each fatty acid was computed as the percentage that a given fatty acid represents relative to the total fatty acid content in the studied type of sample. Then, fatty acids can be classified by the number of double bonds present in the aliphatic chain. Thus, saturated fatty acids (SFA) occur when no double bonds are present, and unsaturated fatty acids (UFA) occur when a single (monounsaturated fatty acids, MUFA) or two or more double bonds (polyunsaturated fatty acids, PUFA) are detected (Guimarães and Venâncio 2022).

##### 2.2.3.2 Determination of Protein and Monosaccharide Content

The Bradford (1976) method was used to determine the soluble protein content (as milligrammes of protein/1 g of sample; [mg/g]). The monosaccharide content (as milligrammes of monosaccharides/1 g of sample; [mg/g]) was estimated according to the Somogyi (1952) method.

### 2.3 Statistical Analysis

The degree of parasitoidism was determined by dividing the number of egg sacs parasitised by the total number of egg sacs collected. The relative reproductive effort (RRE) is expressed as egg sac mass divided by the mass of the corresponding female (Jakob et al. 1996). The results were presented as an arithmetic mean with standard deviation (SD). Statistical analyses included various tests depending on the data being examined. To test the equality of relative reproductive effort (RRE) of *P. lugubris* across half‐month intervals, we used one‐way analysis of variance with Tukey's multiple comparisons test. Similar analyses of *P. lugubris* offspring number and size of egg sacs in subsequent time periods were performed using Welch's test to account for unequal variances, followed by the Games‐Howell multiple comparisons test. The relationship between *P. lugubris* offspring number and the size and the weight of the egg sacs was assessed using the Pearson's linear correlation coefficient (*r*). The statistical significance of the correlation coefficient was checked using a permutation test. The relationship between the presence of pseudoparasitoid in egg sacs of *P. lugubris* and the size of *P. lugubris* females was tested using the Student's *t*‐test. To compare the weight of *P. lugubris* egg sacs with their offspring (separately for eggs, juvenile I and juvenile II) with those containing pseudoparasitoid pupae (spider offspring are absent in these egg sacs), the data were square root transformed to improve normality. Given the persistent heteroscedasticity despite transformation, a Welch's *t*‐test was employed for the analysis. The assumptions of parametric tests were checked using the Shapiro–Wilk test (normality of residual distribution) and Levene's test (homogeneity of variance).

For comparisons of the content of fatty acids, monosaccharides and proteins between *P. lugubris* successive developmental stages in the egg sacs, the nonparametric Kruskal–Wallis test, followed by Dunn's multiple comparison test, was used due to the small sample size within groups. In all analyses, the significance level was set at *p* < 0.05. For the analysis of statistical parameters, Statistica 13 was used (TIBCO Software Inc. (Version 13) 2017).

## 3 Results

### 3.1 The Degree of Parasitoidism of *P. lugubris* Egg Sacs, Along With the Identification and Phenology of the Egg Sac Pseudoparasitoid

The degree of parasitoidism of *P. lugubris* egg sacs ranged from 0% to 27% in different years and populations (Table 1). Only pseudoparasitoids were recorded in the studied egg sacs. All of them were found to be solitary and destroyed the entire content of the egg sacs.

**TABLE 1:** *Hidryta fusiventris* in *P. lugubris* egg sacs in three studied populations in different years. Additional material collected once in the KAT population is also included in the table.

Population	*N* studied egg sacs	*Hidryta fusiventris*			Percentage [%] of parasitised egg sacs
		*N* larvae	*N* pupae	Total	
KAM_2018	107	0	0	0	0.00
KAM_2019	95	0	1	1	1.05
MIC_2019	137	7	30	37	27.01
MIC_2020	190	2	5	7	3.68
MIC_2023	56	5	5	10	17.86
KON_2023	108	8	8	16	14.81
KON_2024	47	1	0	1	2.13
KAT_2023	17	4	5	9	^a^
Total	757	27	54	81	10.70

Abbreviation: *N*, number.

^a^ Not calculated due to obtaining only egg sacs, suspicious about the presence of pseudoparasitoids.

**FIGURE 1:**
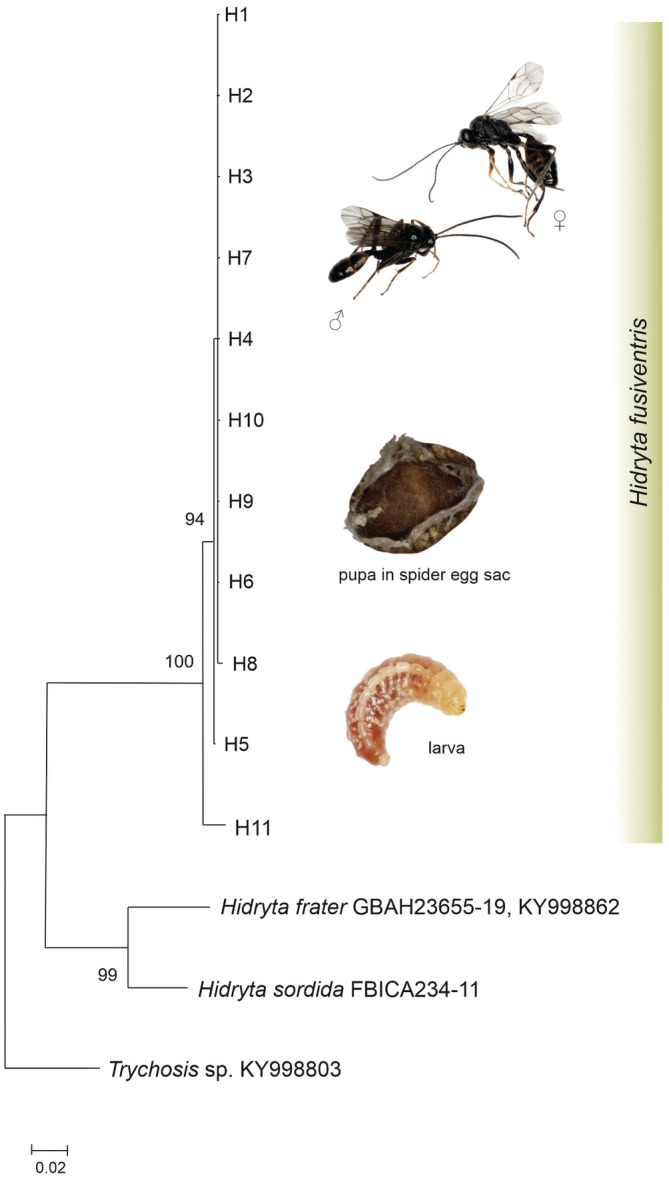
Maximum‐likelihood (ML) phylogenetic tree of the genus *Hidryta* based on mt*COI* sequences (533 bp), including the new haplotypes (H2–H5, H7; see Supporting Information—Table S1, Figure S1) obtained in the present study, as well as sequences available in the BOLD Systems and GeneBank databases (H1, H6, H8–H11). The tree has been rooted with sequences of *Trychosis* sp. Bootstrap values (≥ 70%) are indicated at the nodes. Scale bar indicates branch length in substitutions per site.

Adult specimens of pseudoparasitoids were determined by M. Schwarz as *Hidryta fusiventris* (Thomson, 1873) (Hymenoptera: Ichneumonidae: Cryptinae). This is the first record of the host of this Palaearctic ichneumonid wasp. The maximum likelihood tree (Figure 1) revealed that all haplotypes, obtained from different developmental stages of the studied pseudoparasitoids, created a robust phylogenetic branch (100% bootstrap support) with haplotypes of *H. fusiventris* from other Palaearctic regions. Phylogenetic analyses of 65 sequences of the mt*COI* gene fragment (47 sequences from the studied specimens, and the remaining 18 from databases performed in DnaSP v.5.10 and Arlequin programs have identified 11 haplotypes) (see Table S1, Figure S1), of which seven occurred in *H*. *fusiventris* individuals from Poland (GenBank no. PX508641–PX508647), and out of them, five are new (GenBank no. PX508642–PX508645, PX508645). Phylogenetic analysis of distinct haplotypes clearly distinguished two haplogroups of *H. fusiventris*: the European (H1–H10) and Asian (H11) haplogroups with high bootstrap support on the phylogenetic tree (Figure 1).

**FIGURE 2:**
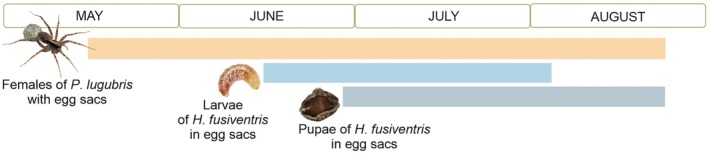
Occurrence of *P. lugubris* females carrying egg sacs and *H*. *fusiventris* in their egg sacs.

*Hidryta fusiventris* larvae were recorded in *P. lugubris* egg sacs from the second part of June until the first days of August, mostly in the first days of July. With one exception at the end of June, the pupae were recorded until about the middle of July (Figure 2). The highest numbers of *H. fusiventris* pupae were observed at the end of July and the beginning of August. Pupae collected in late summer failed in development under laboratory conditions.

### 3.2 Fecundity, RRE, Body Size of *P. lugubris* Females and the Size of Their Egg Sacs

**FIGURE 3:**
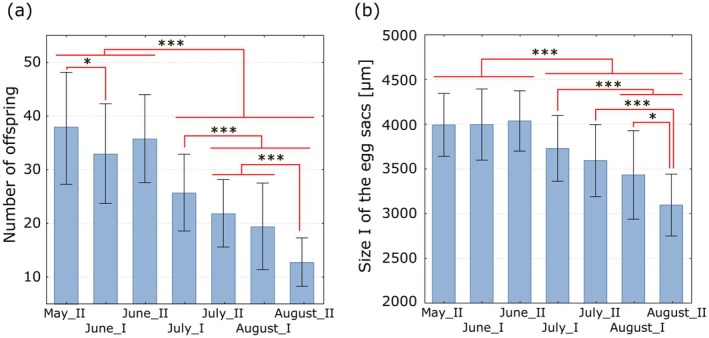
Number of offspring (a) and size I of the egg sacs (b) of *P. lugubris* at half‐month intervals. Blue bars represent the means, whiskers indicate the standard deviations. Red lines show statistical significance in Games‐Howell's multiple comparison test at the significance level: (***) *p* < 0.001; (*) 0.01 < *p* < 0.05.

**FIGURE 4:**
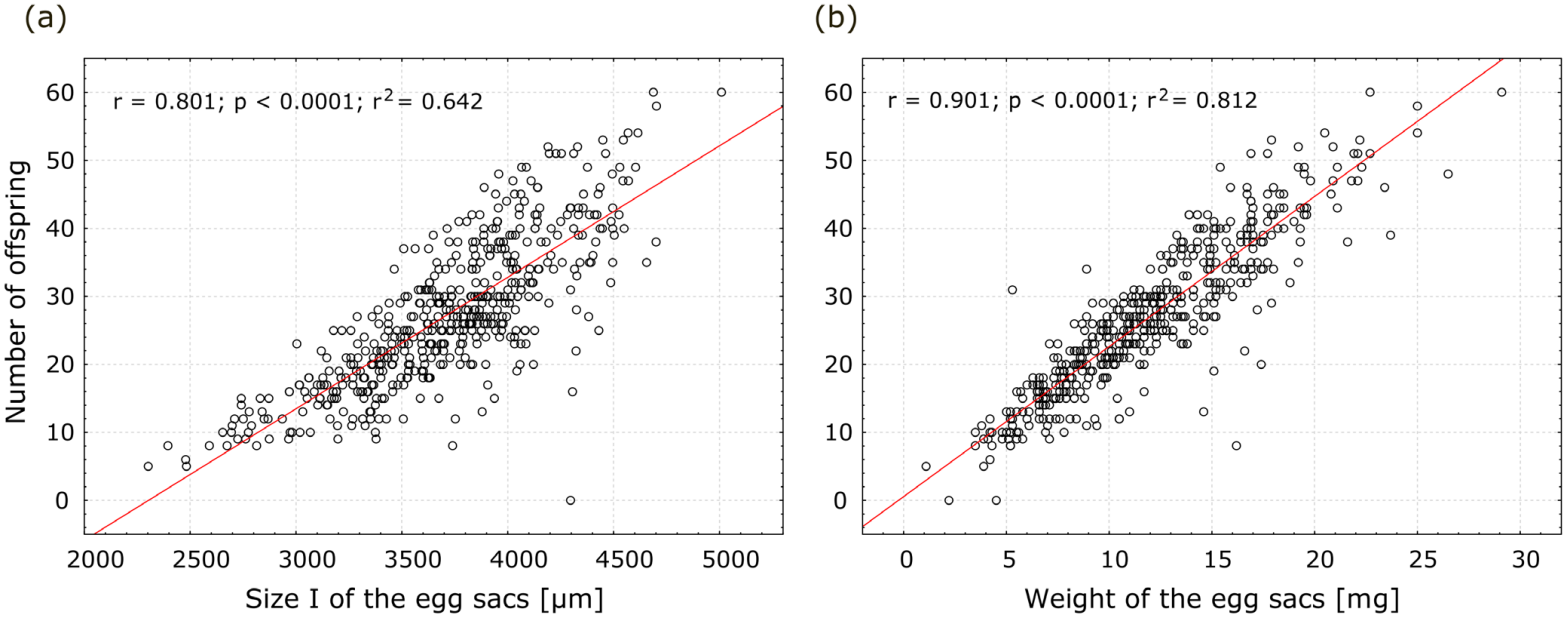
Correlation between the number of *P. lugubris* offspring and the size I (a) and the weight (b) of the egg sacs. The values of Pearson's linear correlation coefficient, along with its significance level and the coefficient of determination, are shown.

The number of spider offspring in the egg sacs did not differ very significantly at the beginning of the reproductive season of *P. lugubris* (from the second part of May to the end of June) but decreased strongly and statistically significantly from July (Figure 3a). The same trend was observed in the size of the egg sacs of *P. lugubris* (Figure 3b). What is more, there was a strong correlation between the size of the egg sac and the number of spider offspring (Figure 4). For the weight of the egg sacs, the Pearson linear correlation coefficient was *r* = 0.9010 and was statistically significant (*p* < 0.0001). The same coefficient was also statistically significant for sizes I and II of the egg sacs, with *r* = 0.801 and *r* = 0.836, respectively.

Analyses of the correlation between female size and egg sac weight and size indicated a weak linear correlation, although statistically significant. The Pearson linear correlation coefficient for the comparison of prosoma width with the size of the egg sacs I and II was *r* = 0.380, *p* < 0.0001 in both cases. When comparing the prosoma's width with the egg sacs' weight, this coefficient was slightly higher and amounted to *r* = 0.433; however, it was still statistically significant (*p* < 0.0001). The relationship between female size and the number of offspring was similar. Analyses revealed a moderate and increasing correlation between the number of offspring and female weight (*r* = 0.446; *p* < 0.0001) and the width of the prosoma (*r* = 0.413; *p* < 0.0001).

**FIGURE 5:**
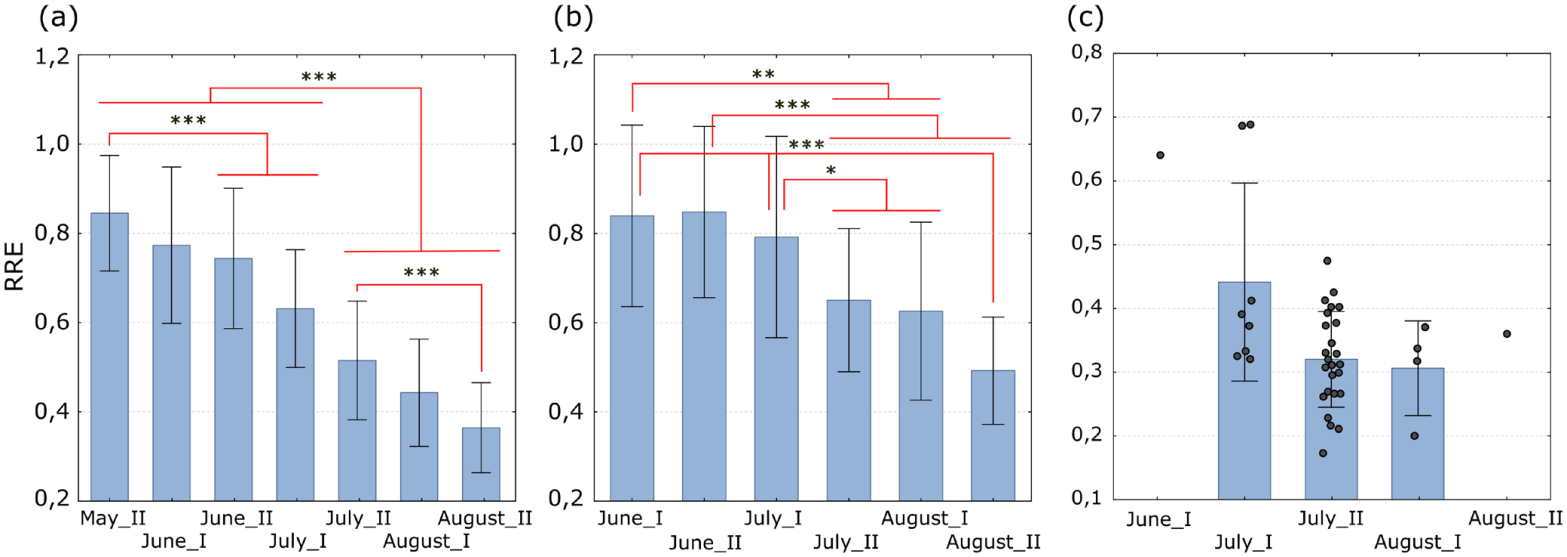
Relative reproductive effort (RRE) of *P. lugubris* at half‐month intervals for females carrying egg sacs with: eggs (a), juveniles (b), pseudoparasitoid pupa (c). Blue bars represent the means, whiskers indicate the standard deviations. The dots in graph c represent the raw data. Red lines show statistical significance in Tukey's HSD test at the significance level: (***) *p* < 0.001; (**) 0.001 < *p* < 0.01; (*) 0.01 < *p* < 0.05.

Relative reproductive effort (RRE) of *P. lugubris* females decreased along with the reproductive season of this spider (Figure 5). In the case of females carrying egg sacs with eggs (Figure 5a) and juveniles (Figure 5b), the greatest differences were recorded between two periods of the reproduction season: (1) the second part of May and June and (2) from the second part of July to the end of August. The number of females carrying pupae was insufficient to conduct reliable analyses; however, the decreasing trend in the RRE value is visible, mainly for the raw data (Figure 5c).

### 3.3 Ovipositional Decision of the *H. fusiventris* Female

**FIGURE 6:**
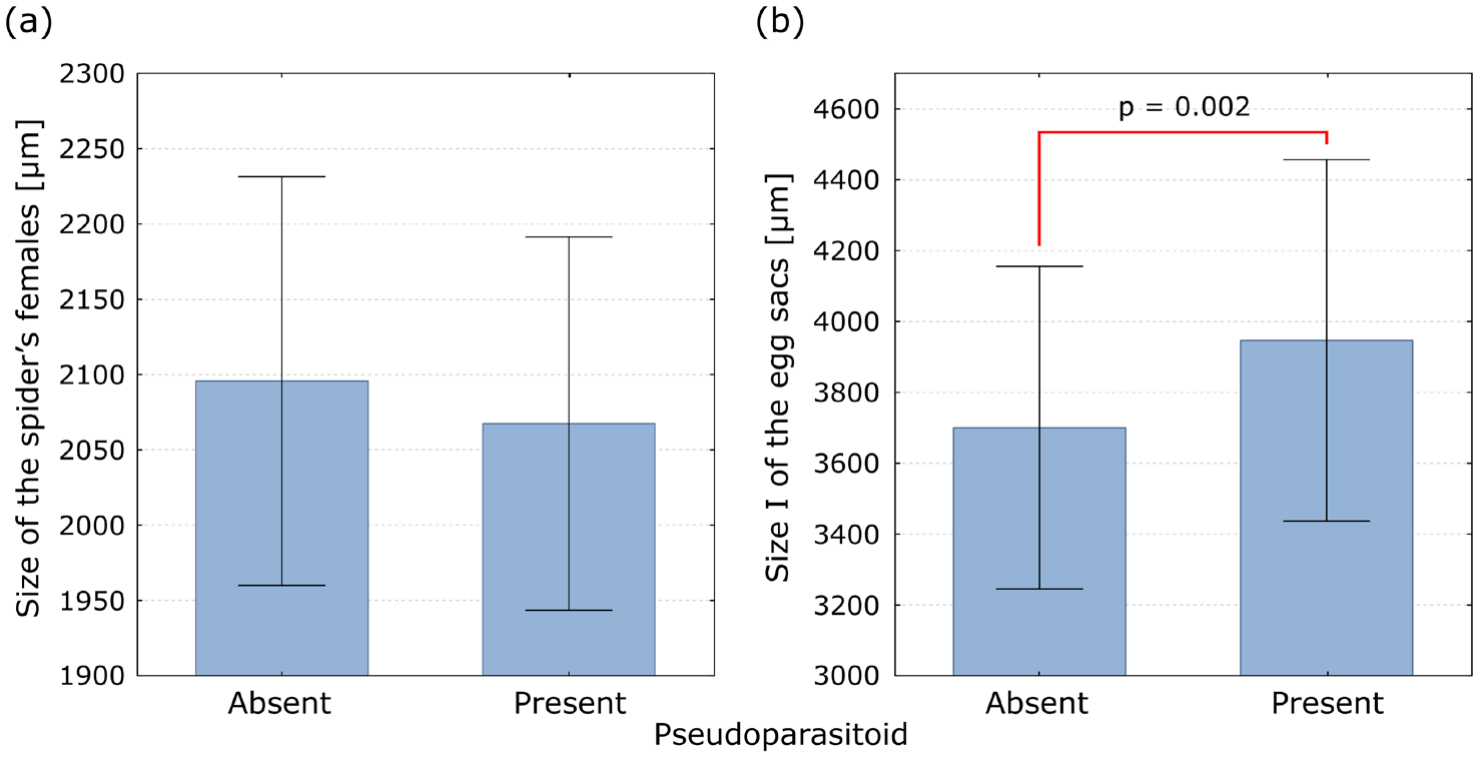
Size (width of prosoma) of the *P. lugubris* females (a) and size I of their egg sacs (b) as cues for the oviposition decision of *H. fusiventris* (pseudoparasitoid present in the spider's egg sac as larva or pupa). Blue bars represent the means, whiskers indicate the standard deviations.

The size of the egg sacs of *P. lugubris* has a significant impact on the presence of the *H*. *fusiventris* in the egg sac (according to the Student's *t*‐test: *p* = 0.002 for the size I egg sacs and *p* < 0.001 for the size II egg sacs), while the body size (width of prosoma) of the *P. lugubris* female has no such effect (*p* = 0.153). It shows that in the case of *H. fusiventris*, female oviposition decision is based on the spider's egg sac size (Figure 6).

### 3.4 *Hidryta fusiventris* Larvae in *P. lugubris* Egg Sacs

**FIGURE 7:**
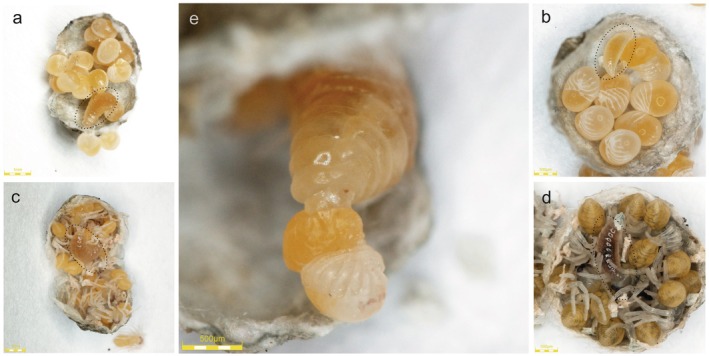
Larva of *H*. *fusiventris* in an egg sac of *P. lugubris* : surrounded by spider eggs and embryos (a), embryos (b), juveniles I (c), juveniles II (d); feeding on spider embryos/juveniles I (e). Larva on photos (a–d) was indicated by dotted ellipse.

We found *H. fusiventris* larvae in 27 of the collected spider egg sacs. The larvae were at various developmental stages and in different egg sacs were surrounded by different stages of spider offspring—eggs, embryos, juvenile Is, or juvenile IIs (Figure 7). Although larvae stopped development after some time, we were able to observe their feeding both on eggs, embryos and on juveniles of *P. lugubris* . In five cases, we observed food preferences of *H. fusiventris* larvae in relation to the developmental stage of spider offspring. In the case where a larva was surrounded by the residue of juvenile II, juveniles I (*n* = 10) of a conspecific spider female were added to the egg sac on the next day after opening the egg sac, and one juvenile was eaten by a pseudoparasitoid. After a day, fresh spider juveniles I (*n* = 5) and eggs (*n* = 5) were added to the egg sac, but the larva ate only one juvenile I and stopped feeding. It was big and very mobile, and the next day it tried to construct a pupal case but unsuccessfully. After the next 10 days, the larva became less mobile, and after the following 13 days, it died. In the four cases in which *H. fusiventris* larvae were surrounded by juvenile I of the spider, larvae fed intensively and grew. After the larvae consumed all the content of the native egg sac, four juveniles I and two eggs from a conspecific egg sac were added—only juveniles I were consumed. Following the cessation of feeding, the *H. fusiventris* larvae produced a few threads; however, none of them were able to make pupal cases. Finally, larvae showed less and less movement, their colour changed to brown, and eventually they died.

### 3.5 *Pardosa lugubris* Maternal Care for Egg Sacs With *H. fusiventris* Pupae

**FIGURE 8:**
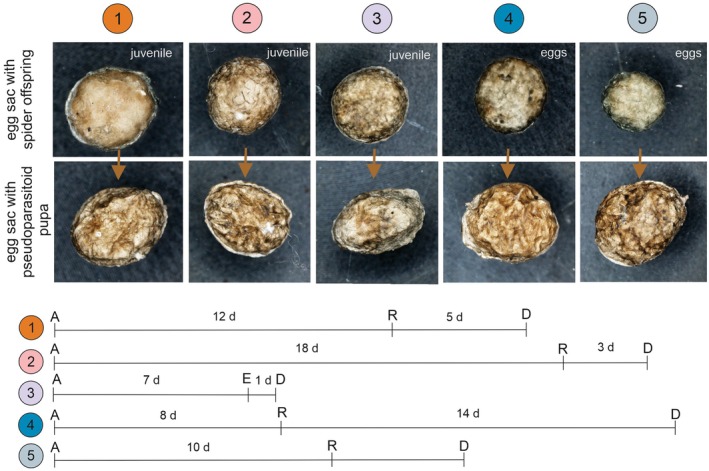
Duration of *P. lugubris* maternal care for a non‐native egg sac with *H*. *fusiventris* pupa (lower line) by a female surrogate spider, which carried her own egg sac with offspring (upper line). A—adoption of non‐native egg sac with pseudoparasitoid pupa, D—death of female spider, E—emergence of pseudoparasitoid imago, R—rejection of egg sac with pseudoparasitoid pupa, d—day(s), 1–6—ordinal number of female spider.

We recorded 54 females of *P. lugubris* that carried egg sacs with *H*. *fusiventris* pupae. These egg sacs were significantly (according to the Welch's *t*‐test: *p* < 0.001) lighter than egg sacs with the spider's offspring, taking into account both eggs, juvenile I and juvenile II. The egg sacs with pseudoparasitoid pupa also had a different shape (more oval) and a more wrinkled outer structure (Figure 8).

In the laboratory, surrogate mothers (*n* = 7) of *P. lugubris* accepted non‐native egg sacs with pseudoparasitoid pupae. They carried these egg sacs for 8–18 days, and after rejection, females lived for several days. In one case, a female spider carried an empty egg sac for a day from after the imago of *H. fusiventris* emerged until her death (Figure 8). Due to unknown reasons, two surrogate females died 3 days after adoption.

Native mothers (*n* = 5) also accepted their own egg sac with pseudoparasitoid pupae and carried them from 6 to 12 days; one died at 6 days while looking after the egg sac. After rejection of the egg sacs with pseudoparasitoid pupae, native females lived 14–15 days.

### 3.6 Content of FAs, Proteins and Monosaccharides in Different Developmental Stages of *P. lugubris* in Egg Sac

**FIGURE 9:**
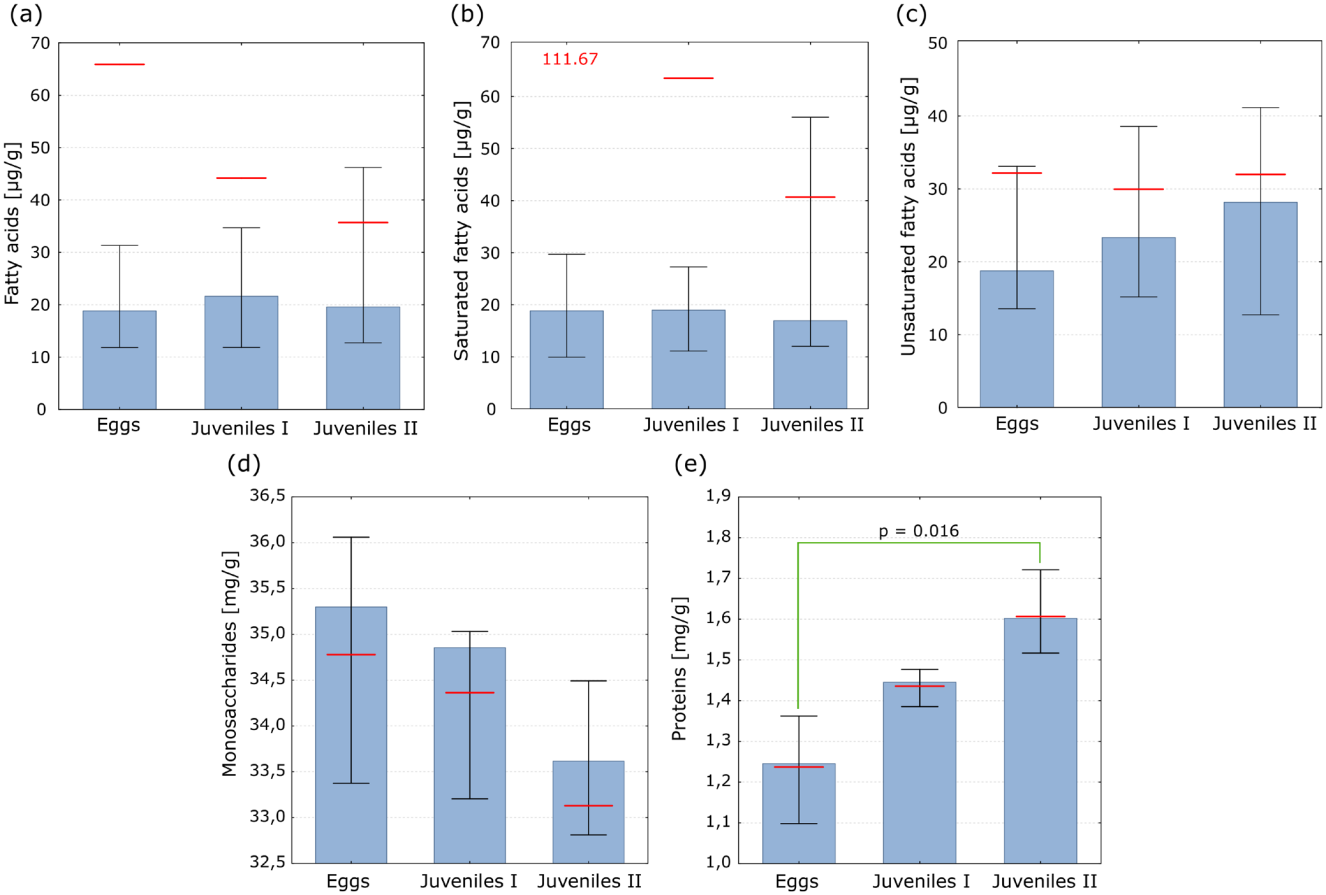
The content of fatty acids (a–c), monosaccharides (d) and proteins (e) in *P. lugubris* successive stages in the egg sac. Blue bars represent the median, whiskers indicate the interquartile range, and the means are marked with red lines/values. Green lines show statistical significance in Dunn's multiple comparison test at a significance level of 0.05.

The eggs, juvenile I and juvenile II of *P. lugubris* included 33 fatty acids (SFA—14, MUFA—9, PUFA—10) from C10:0 to C22:6n3 (Table 2 and Table S2). The total content of FAs, including SFAs, decreased in successive stages (Figure 9a–b), while unsaturated fatty acids (UFA = MUFA + PUFA) were at a similar level (Figure 9c). Although overall differences between subsequent stages were not statistically significant, such differences could be observed for individual FAs, especially between eggs and juvenile IIs. The content of individual fatty acids either increased or decreased depending on the fatty acid (Table 2).

**TABLE 2:** The content [μg/g] of fatty acids in three developmental stages of *P. lugubris* in the egg sac.

Fatty acid	Chain length: no. of double bonds	Eggs		Juvenile I		Juvenile II	
		Mean	SD	Mean	SD	Mean	SD
Capric acid^b^	C10:0	24.61	2.14	21.34	0.60	13.30	1.75
Undecylic acid^b^	C11:0	10.81	1.92	12.47	0.35	17.67	0.84
Lauric acid^b^	C12:0	20.49	2.08	25.93	1.24	56.99	4.15
Tridecylic acid^b^	C13:0	9.31	1.12	11.36	0.32	15.57	1.56
Myristic acid^b^	C14:0	30.88	2.77	27.35	0.77	13.16	1.51
Pentadecylic acid^b^	C15:0	3.17	0.29	2.59	0.53	0.55	0.19
Palmitic acid^b^	C16:0	740.41	37.50	367.26	4.45	200.67	8.66
Margaric acid^b^	C17:0	9.85	1.49	20.59	1.33	28.64	1.89
Stearic acid^b^	C18:0	622.71	55.93	310.10	38.54	111.53	11.19
Arachidic acid^b^	C20:0	32.69	2.63	42.27	1.19	64.11	4.14
Heneicosanoic acid^b^	C21:0	17.40	1.56	17.13	1.85	10.79	0.94
Behenic acid^b^	C22:0	6.82	0.91	9.00	0.93	17.10	0.92
Tricosylic acid^b^	C23:0	20.53	1.66	11.17	0.32	2.13	0.47
Lignoceric acid^b^	C24:0	13.68	1.31	10.73	3.11	17.29	2.53
Myristoleic acid^b^	C14:1	28.35	2.80	39.61	1.12	88.23	3.89
*cis*‐10‐Pentadecenoic acid^b^	C15:1	14.56	3.70	32.81	1.68	56.01	8.80
Palmitoleic acid^b^	C16:1	10.57	1.43	6.82	0.19	5.31	0.65
*cis*‐10‐Heptadecenoic acid^b^	C17:1	3.55	0.31	30.82	2.62	45.82	5.94
Oleic acid^b^	C18:1n9c	29.04	3.90	45.23	1.77	91.60	4.19
Elaidic acid^a^, ^b^	C18:1n9t	27.74	1.94	45.89	1.29	33.91	2.17
Gondoic acid^b^	C20:1	34.96	2.86	21.73	0.61	12.49	0.33
Erucic acid^b^	C22:1n9	112.19	9.62	56.20	10.045	8.61	0.58
Nervonic acid^b^	C24:1n9	13.63	1.24	19.31	0.35	36.37	0.93
Linoleic acid^b^	C18:2n6c	17.66	1.01	22.02	1.16	25.55	2.51
Linolelaidic acid^b^	C18:2n6t	15.39	1.39	23.02	1.85	33.78	5.61
α‐ Linolenic acid^b^	C18:3n3	14.06	3.41	11.89	0.34	7.75	0.83
Gamolenic acid^b^	C18:3n6	47.89	4.86	31.46	2.84	29.62	1.78
*cis*‐11,14‐Eicosadienoic acid^a^, ^b^	C20:2	8.23	0.74	3.04	0.09	3.92	0.54
*cis*‐8,11,14‐Eicosatrienoic acid^a^, ^b^	C20:3n6	14.8419	1.36	8.99	0.25	15.88	2.16
Arachidonic acid^b^	C20:4n6	121.2693	17.89	112.30	3.75	47.88	1.45
Timnodonic acid^b^	C20:5n3	25.6314	2.44	19.27	1.23	18.73	0.52
*cis*‐13,16‐Docasadienoic acid^a^, ^b^	C22:2n6	54.7987	1.95	14.25	1.35	15.25	0.68
Cervonic acid^b^	C22:6n3	16.9924	3.44	24.51	1.26	31.32	5.32
	Total^b^	2174.653	80.53	1458.44	25.46968	1177.40	46.57

*Note:* Statistically significant according to Dunn's multiple comparison test at significance level *p* < 0.05.

Abbreviation: SD, standard deviation.

^a^ Eggs vs. juvenile I.

^b^ Eggs vs. juvenile II.

The relative percentage of saturated to unsaturated FAs was 71.9:28.1, 60.0:39.0 and 48.4:51.6 in eggs, juvenile I and juvenile II, respectively. The FAs with a relatively major percentage in both eggs and juveniles I and II were palmitic acid (C16:0) and stearic acid (C18:0). In juvenile II, the high percentage also included oleic acid (C18:1n9c) and myristoleic acid (C14:1) (Table S2).

The content of proteins increases in the successive developmental stages of *P. lugubris* , but a significant difference was observed only between eggs and juvenile IIs (Figure 9d). The content of monosaccharides noticeably decreases in subsequent spider stages in the egg sac, although differences in medians were not statistically significant (Figure 9e).

## 4 Discussion

### 4.1 Oviposition Decision of *H. fusiventris* Female and Tenable Escape From Pseudoparasitoid by *P. lugubris*

Our study, first carried out on *Hidryta fusiventris*, suggests that like other representatives of the genus *Hidryta*, this ichneumonid wasp is specialised in parasitising the egg sacs of *Pardosa* (Finch 2005). The degree of parasitoidism (0%–27%) of *P. lugubris* egg sacs by *H. fusiventris* shows differences between populations and study years. Despite being lower in certain instances, it is in line with other results of wolf spider egg sac parasitoidism (0%–51.5%) (Bowden and Buddle 2012; Edgar 1971; Koltz et al. 2019). It is probable that during the search for the *P. lugubris* egg sac, *H. fusiventris* responds to the silk odours, as was pointed out for egg sac parasitoids and pseudoparasitoids from other taxa (Eason et al. 1967; Van Baarlen et al. 1996). However, the question is whether in the case of *H*. *fusiventris* oviposition in *P. lugubris* egg sacs is random or there is any selection process. Large hosts are usually assumed to be of higher quality than small hosts because they ensure more resources for larva development (Harvey 2005). In the case of *P. lapponica* and *P. moesta* , egg sac parasitisation by *Gelis* sp. increased significantly with the spider female's body size. Still, this correlation was not shown for the largest of the co‐occurring *Pardosa* species— *P. sodalis* (Bowden and Buddle 2012) or in the study of *P. glacialis* egg sacs parasitised by *Gelis micrurus* (Koltz et al. 2019). In our research, the presence of *H. fusiventris* in spider egg sacs was not correlated with the size of spider females. In contrast, the egg sac size turned out to be a predictor of the oviposition decision of this pseudoparasitoid that corresponds to what we expected in our hypothesis (H: 1). In the case of spiders that produce more than one egg sac per year, the size of the egg sac seems to be a better cue for the pseudoparasitoid female to oviposit than the size of the spider female. In the vast majority of cases, the spider's first egg sac is the largest, with successive clutches declining monotonically in size (Marshall and Gittleman 1994). In the case of *P. lugubris* , we showed that the size of the egg sacs and the number of offspring in them did not differ at the beginning of its reproductive season (from the second part of May to the end of June, probably when the first egg sacs are done), but decreased significantly from July. However, we observed a time shift of approximately 1 month between the recording of the first egg sacs of *P. lugubris* and the presence of larvae of *H. fusiventris* in the egg sacs. The relative reproductive effort of *P. lugubris* females is highest at the beginning of the season—the egg sacs contain more eggs and are heavier than later egg sacs. Therefore, avoiding pseudoparasitoids during this time has the most significant impact on the fitness of *P. lugubris* females, especially due to the fact that *H. fusiventris* larva destroys the entire content of the egg sac in which it develops. We think that the observed time shift in the phenology of the studied species is the kind of tenable escape of *P. lugubris* from the pseudoparasitoid. This kind of escape was also suggested in *Argiope bruennichi* (Scopoli, 1772). Because only egg sacs with eggs are parasitised by *Tromatobia ornata* (Gravenhorst, 1829) (Rollard 1987), females of *A. bruennichi* that lay their eggs early have an advantage, as their offspring can hatch before the pseudoparasitoid oviposits (Leborgne and Pasquet 2005).

### 4.2 Feeding of *H*. *fusiventris* Larva in *P. lugubris* Egg Sac

In general, it is assumed that pseudoparasitoid larvae developing in the egg sac feed on the spider's eggs (Edgar 1971; Iwata 1963; Rollard 1990; Souza‐Santiago et al. 2023; Takasuka and Broad 2024; Villanueva‐Bonilla et al. 2016). The ichneumonid wasp *Tromatobia ovivora* (Boheman, 1821), before parasitising the egg sacs of *Araneus diadematus* Clerck, 1758 or *A. quadratus* Clerck, 1757, inspects them and rejects all egg sacs other than those containing unhatched and unparasitised egg masses (Crome 1959). This inspection and the ability to discriminate between hatched or unhatched eggs was also observed in *T. ornata* parasitising on an *Argiope bruennichi* egg sac or *Zaglyptus iwatai* (Uchida, 1936), which oviposits in *Clubiona japonicola* Bösenberg & Strand, 1906 egg mass (Iwata 1963). In the latter case, ovipositing in egg mass which was not fresh led to a lack of food for the pseudoparasitoid larvae because the *Z. iwatai* larvae do not feed on the post‐hatching offspring of the spider (Iwata 1963).

Due to the active maternal care of egg sacs and active hunting by wolf spiders, ovipositing in their egg sacs appears exceedingly challenging for pseudoparasitoids. Spider maternal care prevents pseudoparasitoid females from engaging in leisurely oviposition behaviour with its careful inspections. We expected a trade‐off between the quick oviposition of a single egg without inspecting the content of the guarded egg sac, which can lead to selecting an egg sac with inappropriate content for pseudoparasitoid larva, the avoidance of interaction with the spider, survival, and, in turn, potential for oviposition in the next egg sacs of numerous *Pardosa* females. Indeed, larvae of *H. fusiventris* were found in the surroundings of different developmental stages of *P. lugubris* , but interestingly, the pseudoparasitoid larvae were able to feed on all of them. These results are consistent with our hypothesis (H: 2) and observation made on *Trychosis* sp. larvae developing in *Pisauria mirabilis* (Clerck, 1757) egg sacs, carried by the female in chelicerae (Austad and Thornhill 1986). Is feeding on pre‐ and post‐hatched stages of spiders in egg sacs an adaptation of pseudoparasitoids that oviposit in actively guarded spider egg sacs? This ability certainly enables the exploitation of long‐term food resources, not limited to a specific stage. On the other hand, because the chemical composition of the successive developmental stages of the spider in the egg sac changes over time (Laino et al. 2013; Romero et al. 2022; Suprunowicz, Kostro‐Ambroziak, et al. 2025; Trabalon et al. 2018), it may affect the development of *H. fusiventris* and its fitness‐related traits. The content of fatty acids (mainly saturated FAs) and monosaccharides decreased through the developmental stages of *P. lugubris* . Significant lipid, protein and carbohydrate depletion from the undivided egg to the emerged offspring was also shown in *Pardosa saltans* Töpfer‐Hofmann, 2000 and *Polybetes pythagoricus* (Holmberg, 1875) (Romero et al. 2022; Trabalon et al. 2018). Thus, the most valuable food source for pseudoparasitoid larvae developing in egg sacs seems to be spider eggs that is in line with what we expected in our hypothesis (H: 3). It is especially vital in terms of lipid accumulation in the larval stage, primarily in the insect's fat body, which provides the adult with a long‐term energy reserve and is a good predictor of adult longevity and fecundity (Ellers 1996; Scheifler et al. 2024).

The content of each recorded FA changed significantly between eggs and juvenile IIs of *P. lugubris* but, depending on the compound, it increased or decreased. This is especially important because parasitoids (including ichneumonid wasps) incorporate host‐derived fatty acids directly into their own fat stores rather than synthesising them *de novo* (Scheifler et al. 2024). Additionally, despite the fact that some ichneumonid wasps can synthesise FAs (Ruther et al. 2021), they, like other insects, cannot synthesise essential fatty acids (EFAs) (such as linoleic acid and α‐linolenic acid), which are vital for proper growth and development, serving as the building blocks to make other ω3 and ω6 families of long‐chain polyunsaturated fatty acids. We observed depletion of α‐linolenic acid (C18:3n3s) but an increase in linoleic acid (C18:2n6c) from eggs to juvenile IIs of *P. lugubris* . One of the main derivatives of linoleic acid is arachidonic acid (20:4ω6), which is crucial for various physiological functions, such as the structure and signalling of the central nervous system, defensive immune reactions, hatching and moulting (Dadd and Kleinjan 1979; Tallima and El Ridi 2018; Wen et al. 2025). Arachidonic acid is one of the dominant FAs in *P. lugubris* eggs, which is consistent with the finding that lycosids have the highest content of this FA compared with other spider families (Ramberg et al. 2020). The content of arachidonic acid is more or less similar in eggs and juvenile Is, but significantly decreases in juvenile IIs, a decrease which is balanced by an increase in linoleic acid. Thus, it is not clear what the impact is on the pseudoparasitoid of feeding on other spider stages than eggs, when we consider not only the total content of main chemical groups but also their quantity and quality composition.

### 4.3 *Pardosa lugubris* Maternal Care for Egg Sacs With *H*. *fusiventris* Pupae

The maternal care exhibited by *Pardosa* females incurs an energetic cost and the risk of predation (Colancecco and Rypstra 2007; Ruhland et al. 2016). In the case of the presence of the pseudoparasitoid in the egg sac, it would be a huge advantage for a female spider to abandon these egg sacs and make new ones. We found over 50 females of *P. lugubris* carrying egg sacs with *H*. *fusiventris* pupa in the field, showing that the females of *P. lugubris* continue to care for these egg sacs. The egg sac of *P. lugubris* with pupa of *H. fusiventris* acquired a different shape in relation to the circular egg sac with spider offspring—it was more oval due to the presence of a big, hard pupal case with the pseudoparasitoid in it. Similar changes were also observed in the presence of *Hidryta sordida* in the egg sac of *P. lugubris* (Edgar 1971). In the opposite of what we hypothesised (H: 4), the egg sacs with *H. fusiventris* pupae were also significantly lighter than egg sacs with spider offspring. Ruhland et al. (2019) showed that females of *P. saltans* discriminated between egg sacs differing in mass (empty or not) and terminated care for empty egg sacs. The weight of *P. lugubris* egg sacs decreased along with the reproductive season of this spider. We assume this may be an advantage for *H*. *fusiventris*, as its pupa was not recorded until the second part of the reproductive season of *P. lugubris* . Thus, it is probable that the spider females do not feel a big difference between an egg sac with their own offspring and an egg sac with a pseudoparasitoid pupa. Furthermore, it was shown that abandonment of the second egg sac of *Pardosa milvina* (Hentz, 1844) decreased, which is in line with the predictions of life history theory that indicate that, along with diminishing future reproductive potential, parents should maximise parental care and be less sensitive to the viability of offspring (Marchetti and Persons 2020). Although the vibration emitted by juveniles and chemical signals are involved in egg sac care in *P. saltans* , it was shown that females of this spider do not discriminate between egg sacs with viable and nonviable offspring, and continue taking care of them until the predicted date of offspring emergence from the egg sac (about a 30‐day‐old egg sac) (Ruhland et al. 2019).

Interestingly, *P. lugubris* females accepted an egg sac with a pseudoparasitoid pupa from conspecific females independently of their own egg sac age and content. Adoption of an unfamiliar egg sac from conspecific females, but with spider's offspring, was also recorded in *P. milvina* and *P. saltans* (Culley et al. 2010; Ruhland et al. 2017). Female wolf spiders use composite information (size, texture, shape, colour, odour) rather than a single cue to recognise their egg sac, but chemical signals seem to be the most important in modifying the behavioural activities of *Pardosa* females (Culley et al. 2010; Ruhland et al. 2016; Suprunowicz, Kostro‐Ambroziak, et al. 2025). Since there are no *P. lugubris* offspring in the egg sac with *H*. *fusiventris* pupa, and the shape, weight, toughness and texture of this egg sac are changed, we assume that *H*. *fusiventris* uses some form of chemical camouflage (mimicry), as was suggested in the case of *Tromatobia* sp. observed in the egg sac of cobweb spider *Chrysso compressa* (Eugen von Keyserling, 1884) (Souza‐Santiago et al. 2023). Although there are still many questions about ‘why’, the data from the field and behavioural observations in the laboratory showed that *P. lugubris* females do not reject egg sacs with *H*. *fusiventris* pupa, which corresponds to what we expected in our hypothesis (H: 5).

### 4.4 Spider Care and Pseudoparasitoid Survival

All the larvae of *H*. *fusiventris* reared in the laboratory in the open egg sacs of *P. lugubris* died at various stages of development. It is known that the inner surface of the egg sac of *Parasteatoda tepidariorum* (C. L. Koch, 1841) is sterile (Babczyńska et al. 2019). The chemical composition of spider egg sac silks and eggs (Glenszczyk et al. 2025; Suprunowicz, Piotrowska‐Niczyporuk, and Kostro‐Ambroziak 2025) suggests that antimicrobial mechanisms are likely present in the egg sacs of other spiders. We presume that this antimicrobial protection is also crucial for pseudoparasitoid development. Furthermore, the egg sac and the spider female's active care ensure proper humidity and temperature within the egg sac for spider offspring (Austin 1985; Foelix 2025), which may be no less important for the *H*. *fusiventris* larvae. Furthermore, although we observed the initial stages of the pupal case‐making process, they ultimately failed. We think that a closed egg sac is needed to sustain the construction of a pseudoparasitoid pupal case. Moreover, no adult pseudoparasitoids emerged from many of the egg sacs with pupae of *H*. *fusiventris*, which were detached from *P. lugubris* , even though the egg sacs were not opened. Hence, the various aspects of maternal spider care on pseudoparasitoid survival should be investigated in future studies.

## 5 Conclusion

Our study provided important new insights into the evolutionary arms race between spiders that exhibit active maternal care of egg sacs and egg sac pseudoparasitoids. All stated hypotheses were verified, showing many adaptations in both components of this relationship and, at the same time, indicating that many questions still remain unanswered. We recorded that *P. lugubris* females show tenable escape from *H*. *fusiventris* at the beginning of their reproductive season, when their relative reproductive effort is the highest. However, the oviposition decision of *H. fusiventris* based on the size of the egg sac leads to the choice of the best from the present egg sacs at a given time. Furthermore, *H*. *fusiventris*'s ability to feed on all *P. lugubris* developmental stages in egg sacs is a good adaptation, especially important when ovipositing in actively guarded spider egg sacs, which do not allow leisurely oviposition behaviour with its careful choosing of appropriate egg sac content. However, changes in chemical composition throughout the developmental stages of *P. lugubris* may affect the development of *H*. *fusiventris* and its fitness‐related traits. Does *H*. *fusiventris* exhibit a plasticity in fatty acid synthesis that depends on the quality of the host? We showed that *P. lugubris* does not reject egg sacs with pseudoparasitoid pupa, investing in the care of alien offspring. We do not know the distinct reason. Do *H*. *fusiventris* larva/pupae exhibit some kind of chemical camouflage? We assumed that spider care, probably for many reasons, is necessary for the proper development of *H*. *fusiventris* in the egg sac. Is it true? Many points in the studied area need further investigation.

## Author Contributions

**Agata Kostro‐Ambroziak:** conceptualization (lead), data curation (equal), formal analysis (equal), investigation (equal), methodology (equal), resources (equal), supervision (lead), visualization (equal), writing – original draft (lead). **Urszula Suprunowicz:** conceptualization (supporting), data curation (lead), formal analysis (equal), investigation (equal), methodology (equal), writing – original draft (supporting). **Alicja Piotrowska‐Niczyporuk:** data curation (supporting), formal analysis (equal), methodology (equal), writing – original draft (supporting). **Magdalena Czajkowska:** formal analysis (equal), methodology (equal), visualization (supporting), writing – original draft (supporting). **Elżbieta Sandurska:** formal analysis (equal), methodology (equal), visualization (equal), writing – original draft (supporting). **Dawid Szymański:** investigation (equal). **Dominik Szymański:** investigation (equal).

## Funding

This article has received financial support from the Polish Ministry of Science and Higher Education, under the subsidy for maintaining the research potential of the Faculty of Biology, University of Bialystok.

## Conflicts of Interest

The authors declare no conflicts of interest.

## Supporting information


**Figure S1:** Haplotype network of *Hidryta fusiventris*. Haplotype networks were constructed using the median‐joining method implemented in Network v.10.2.0.0 software (https://www.fluxus‐engineering.com). Each node size reflects the frequency of a particular haplotype, and node colour corresponds to the origin. The lengths of the lines are proportional to genetic distance.
**Table S1:** Frequencies of 11 haplotypes of the 533 bp COI mitochondrial gene identified in four populations of *Hidryta fusiventris* from Poland (7 haplotypes: H1–H7) and deposited under GenBank accession number: PX508641–PX508647.
**Table S2:** Composition of fatty acids and relative proportions [%] of each fatty acid and the main group of fatty acids in three developmental stages of *P. lugubris* in the egg sac.

## Data Availability

All relevant data are included in the manuscript and its Supporting Information files. The genetic data generated during the current study are deposited in GenBank under accession number: PX508641–PX508647.
