# Supplementary material for: New Insight Into the Evolutionary Arms Race Between Spider Egg Sac Pseudoparasitoids and Active Maternal Care by the Spiders
Source: Ecol Evol. 2026 Apr 30;16(5):e73581. doi: 10.1002/ece3.73581 (PMC13129604; doi:10.1002/ece3.73581)
Supplement: Supplementary file 1 — Figure S1: Haplotype network of Hidryta fusiventris. Haplotype networks were constructed using the median‐joining method implemented in Network v.10.2.0.0 software (https://www.fluxus‐engineering.com). Each node size reflects the frequency of a particular haplotype, and node colour corresponds to the origin. The lengths of the lines are proportional to genetic distance. Table S1: Frequencies of 11 haplotypes of the 533 bp COI mitochondrial gene identified in four populations of Hidryta fusiventris from Poland (7 haplotypes: H1–H7) and deposited under GenBank accession number: PX508641–PX508647. Table S2: Composition of fatty acids and relative proportions [%] of each fatty acid and the main group of fatty acids in three developmental stages of P. lugubris in the egg sac. [file ECE3-16-e73581-s001.pdf]

New insight into the evolutionary arms race between spider egg sac pseudoparasitoids and active maternal care by the spiders

Supporting Information

Table S1. Frequencies of 11 haplotypes of the 533 bp COI mitochondrial gene identified in four populations of *Hidryta fusiventris* from Poland (7 haplotypes: H1–H7) and deposited under GenBank accession number: PX508641-PX508647.

Haplotype \ Population	MIC (N=33)	KON (N=9)	KAT (N=4)	KAM (N=1)	DE (N=14)	AT (N=1)	NO (N=2)	RU (N=1)
H1	0.67	0.67	1.00	-	0.65	1.00	0.50	-
H2	0.18	-	-	-	-	-	-	-
H3	0.03	-	-	-	-	-	-	-
H4	0.12	-	-	-	-	-	-	-
H5	-	0.22	-	-	-	-	-	-
H6	-	0.11	-	-	-	-	0.50	-
H7	-	-	-	1.00	-	-	-	-
H8	-	-	-	-	0.07	-	-	-
H9	-	-	-	-	0.14	-	-	-
H10	-	-	-	-	0.14	-	-	-
H11	-	-	-	-	-	-	-	1.00

Population symbols (numbers of sequences used from the BOLD Systems and GenBank database are given in brackets): MIC – Poland1; KON – Poland2; KAT – Poland3; KAM – Poland4; DE – Germany (GMGMA758-14. GMGMA968-14. GMGMA1025-14. GMGMI1399-14. GMGMJ1314-14. GMGML374-14. GMGML634-14. GMGML699-14. GMGML1477-14. GMGMN552-14. GMGRF3413-13. GMGRF5473-13. MT302536. FBICA235-11); NO – Norway (NOPRA977-17. HYMPK072-23); AT – Austria (FBICA235-11); RU – Russian Federation (GMRSW096-15).

H1: Poland (GenBank no. PX508641), Germany (BOLD no. GMGMA758-14, GMGMA968-14, GMGMC736-14, GMGML1477-14, GMGMJ1314-14, GMGMI1399-14, GMGMN552-14, GMGRF3413-13, GMGRF5473-13), Austria (BOLD no. FBICA235-11), Norway (BOLD no. HYMPK072-23); **H2**: Poland (GenBank no. PX508642); **H3**: Poland (GenBank no. PX508643); **H4**: Poland (GenBank no. PX508644); **H5**: Poland (GenBank no. PX508645); **H6**: Poland (GenBank no. PX508646), Norway (BOLD no. NOPRA977-17); **H7**: Poland (GenBank no. PX508647); **H8**: Germany (GenBank no. MT302536); **H9**: Germany (BOLD no. GMGMA1025-14, GMGML374-14); **H10**: Germany (BOLD no. GMGML699-14, GMGML634-14); **H11**: Russia (BOLD no. GMRSW096-15).

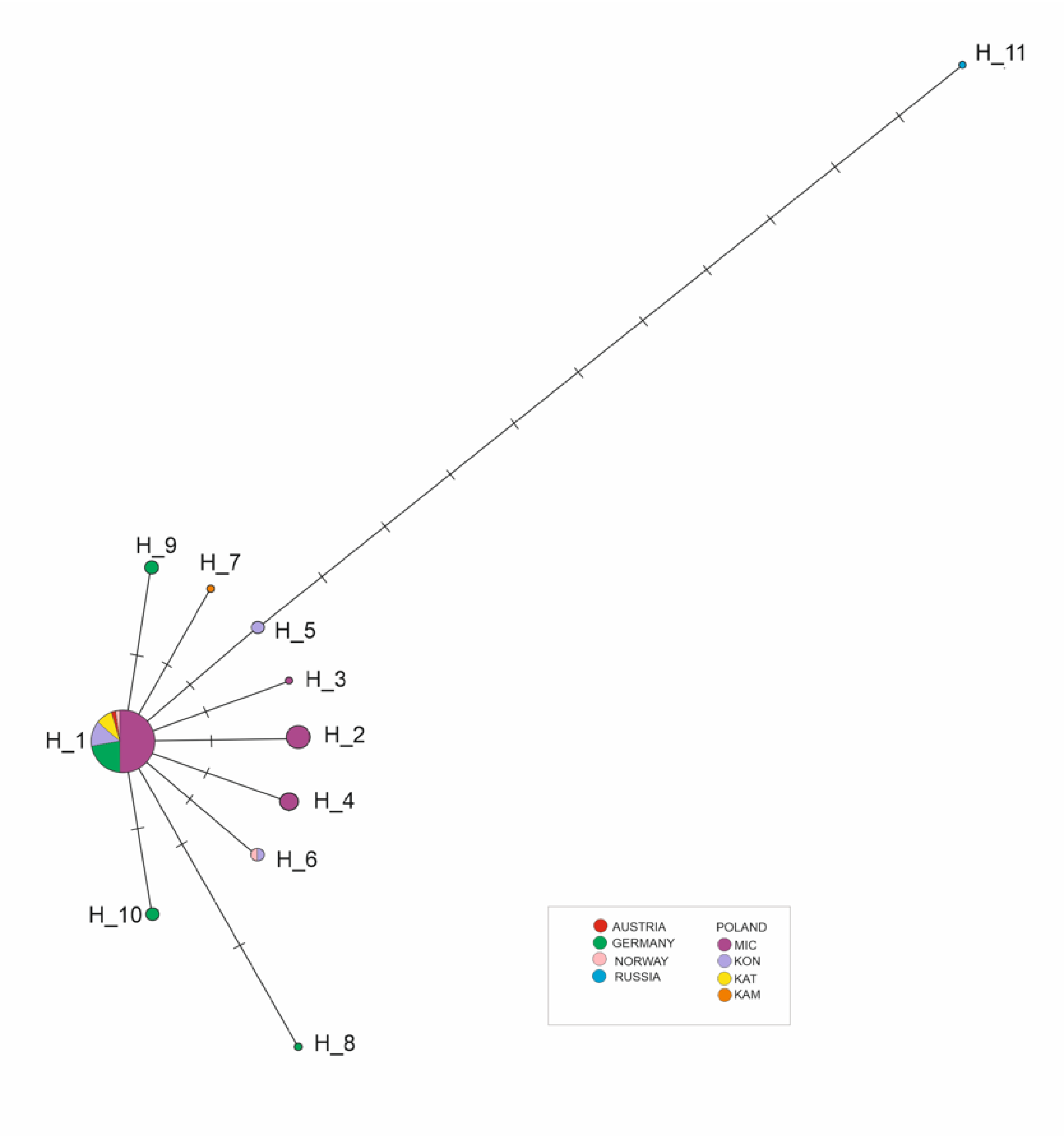

Figure S1. Haplotype network of *Hidryta fusiventris*. Haplotype networks were constructed using the median-joining method implemented in Network v.10.2.0.0 software (https://www.fluxus-engineering.com). Each node size reflects the frequency of a particular haplotype, and node colour corresponds to the origin. The lengths of the lines are proportional to genetic distance.

Table S2. Composition of fatty acids and relative proportions [%] of each fatty acid and the main group of fatty acids in three developmental stages of *P. lugubris* in the egg sac.

Fatty acid	Chain length: no. of double bonds	Eggs	Juvenile I	Juvenile II
Capric acid	C10:0	1.13	1.46	1.13
Undecylic acid	C11:0	0.50	0.86	1.50
Lauric acid	C12:0	0.94	1.78	4.84
Tridecylic acid	C13:0	0.43	0.78	1.32
Myristic acid	C14:0	1.42	1.88	1.12
Pentadecylic acid	C15:0	0.15	0.18	0.05
Palmitic acid	C16:0	34.05	25.18	17.04
Margaric acid	C17:0	0.45	1.41	2.43
Stearic acid	C18:0	28.64	21.26	9.47
Arachidic acid	C20:0	1.50	2.90	5.45
Heneicosanoic acid	C21:0	0.80	1.18	0.91
Behenic acid	C22:0	0.31	0.62	1.44
Tricosylic acid	C23:0	0.94	0.77	0.18
Lignoceric acid	C24:0	0.63	0.74	1.47
Myristoleic acid	C14:1	1.30	2.72	7.49
cis-10-Pentadecenoic acid	C15:1	0.67	2.25	4.76
Palmitoleic acid	C16:1	0.49	0.47	0.45
cis-10-Heptadecenoic acid	C17:1	0.16	2.11	3.89
Oleic acid	C18:1n9c	1.34	3.10	7.78
Elaidic acid	C18:1n9t	1.28	3.15	2.88
Gondoic acid	C20:1	1.61	1.49	1.06
Erucic acid	C22:1n9	5.16	3.85	0.73
Nervonic acid	C24:1n9	0.63	1.32	3.09
Linoleic acid	C18:2n6c	0.81	1.51	2.17
Linolelaidic acid	C18:2n6t	0.71	1.58	2.87
Linolenic acid	C18:3n3	0.65	0.82	0.66
Gamolenic acid	C18:3n6	2.20	2.16	2.52
cis-11.14-Eicosadienoic acid	C20:2	0.38	0.21	0.33
cis-8.11.14-Eicosatrienoic acid	C20:3n6	0.68	0.62	1.35
Arachidonic acid	C20:4n6	5.58	7.70	4.07
Timnodonic acid	C20:5n3	1.18	1.32	1.59
cis-13.16-Docasadienoic acid	C22:2n6	2.52	0.98	1.26
Cervonic acid	C22:6n3	0.78	1.68	2.66
SFA		71.89	60.98	48.35
MUFA		12.63	20.46	32.14
PUFA		15.48	18.56	19.51
UFA		28.11	39.02	51.62

SFA – saturated fatty acids (SFA). MUFA – monounsaturated fatty acids. PUFA – polyunsaturated fatty acids, UFA (MUFA+PUFA) – unsaturated fatty acids
